# Disseminated plasma cell myeloma presenting as massive pleural effusion

**DOI:** 10.3402/ecrj.v2.27028

**Published:** 2015-11-24

**Authors:** Kanahasubramanian Anand Babu, Lakshmikanthan Sundararajan, Pandurangan Prabu, Ashok Parameswaran

**Affiliations:** 1 Respiratory Medicine, Apollo Hospitals, Chennai, India; 2 Haematology, Apollo Hospitals, Chennai, India; 3 Department of Pathology, Apollo Hospital, Chennai, India

**Keywords:** Plasma cell myeloma, myelomatous pleural effusion, pleural fluid cytology, massive pleural effusion

## Abstract

Plasma cell myeloma (PCM) is a hematologic malignancy of plasma cell origin and usually associated with the presence of lytic bone lesions. Pleural effusions are rarely associated with PCM and most often signify a concurrent disease process. Malignant myelomatous pleural effusions are even more unusual and carry a poor prognosis. We report a unique case of unsuspected PCM with thoracic involvement in the form of massive left side pleural effusion. Pleural fluid cytology revealed numerous atypical plasma cells. Subsequently on further workup, urine Bence Jones protein was positive. Bone marrow aspiration and biopsy and computed tomography of the chest and abdomen revealed features consistent with multiple myeloma.

Pleural effusion is a relatively uncommon finding in myeloma patients, with a frequency of only 6%, and is usually caused by nephrotic syndrome, pulmonary embolism, congestive heart failure secondary to amyloidosis, and infection. Myelomatous pleural effusion is extremely rare, occurring in less than 1% of cases, and seldom is a presenting feature (1). Pleural effusion in plasma cell myeloma (PCM) usually indicates a poor prognosis for myeloma with mean survival less than 4 months (2). Here we report an extremely rare case presenting with multiple thoracic manifestations in whom a diagnosis of PCM was established after a thorough investigation. Computed tomography (CT) scan of the chest and abdomen revealed extensive involvement of pleura, lung parenchyma, mediastinum, retroperitoneum, bone, and soft tissue. The rarity of this pulmonary manifestation of plasma cell myeloma prompted this report.

## Case report

A 48-year-old man from North East India presented to the emergency department of our hospital with breathlessness, pleuritic chest pain and dry cough for 2 months. He had no history of fever, hemoptysis, joint pain, or palpitations. There was no significant past medical history. Initially he was evaluated in another hospital and diagnosed to have a left-sided pleural effusion on chest X-ray. Pleural fluid aspiration was done, and it was reported as hemorrhagic fluid, details of which were not available. On admission to our hospital, on physical examination he was in respiratory distress and hypoxic, with an oxygen saturation of 86% on room air. Hemodynamically he was stable. Chest examination revealed absent breath sounds in the left hemithorax, with dullness on percussion. The abdomen was soft, no organomegaly, no pedal edema, and cardiac vascular system examination was normal.

**Fig. 1:**
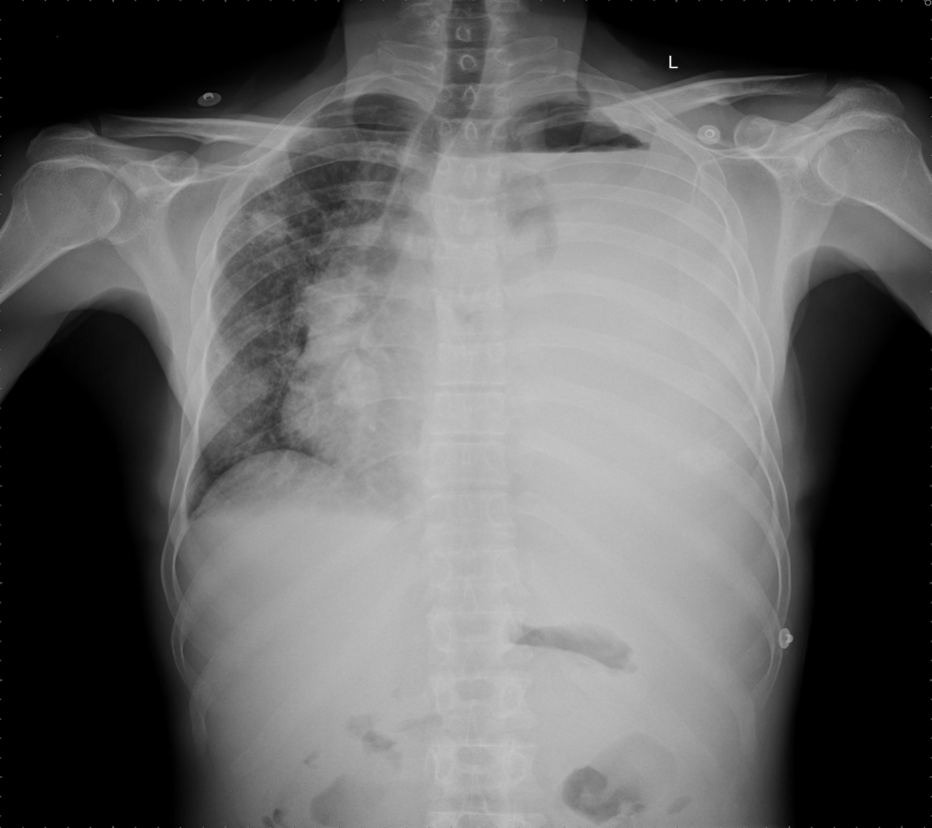
Chest X-ray showing massive left side pleural effusion and right upper zone nodular opacity (small air pocket in left apex is due to previous aspiration).

**Fig. 2:**
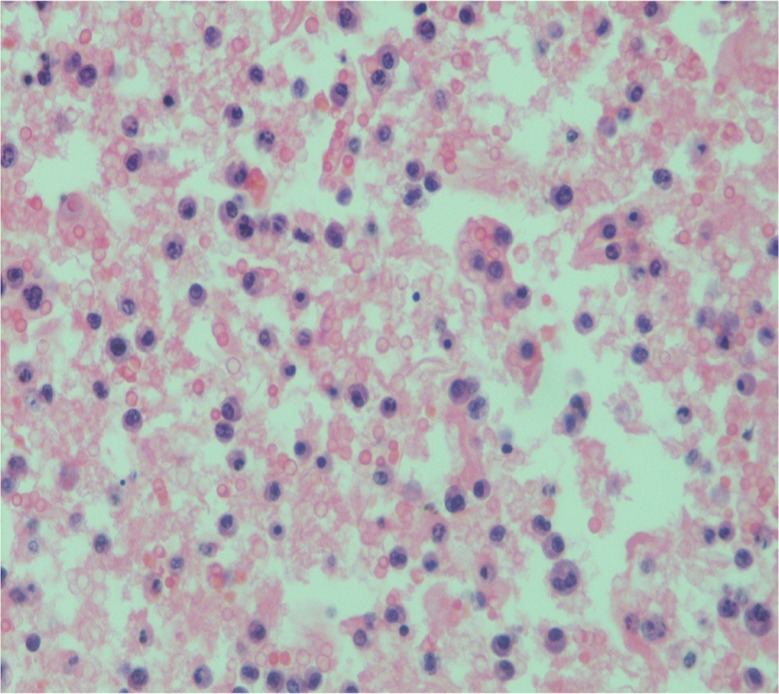
Cytological examination of the pleural fluid showing numerous atypical plasma cells with binucleate forms and plasmablasts.

**Fig. 3:**
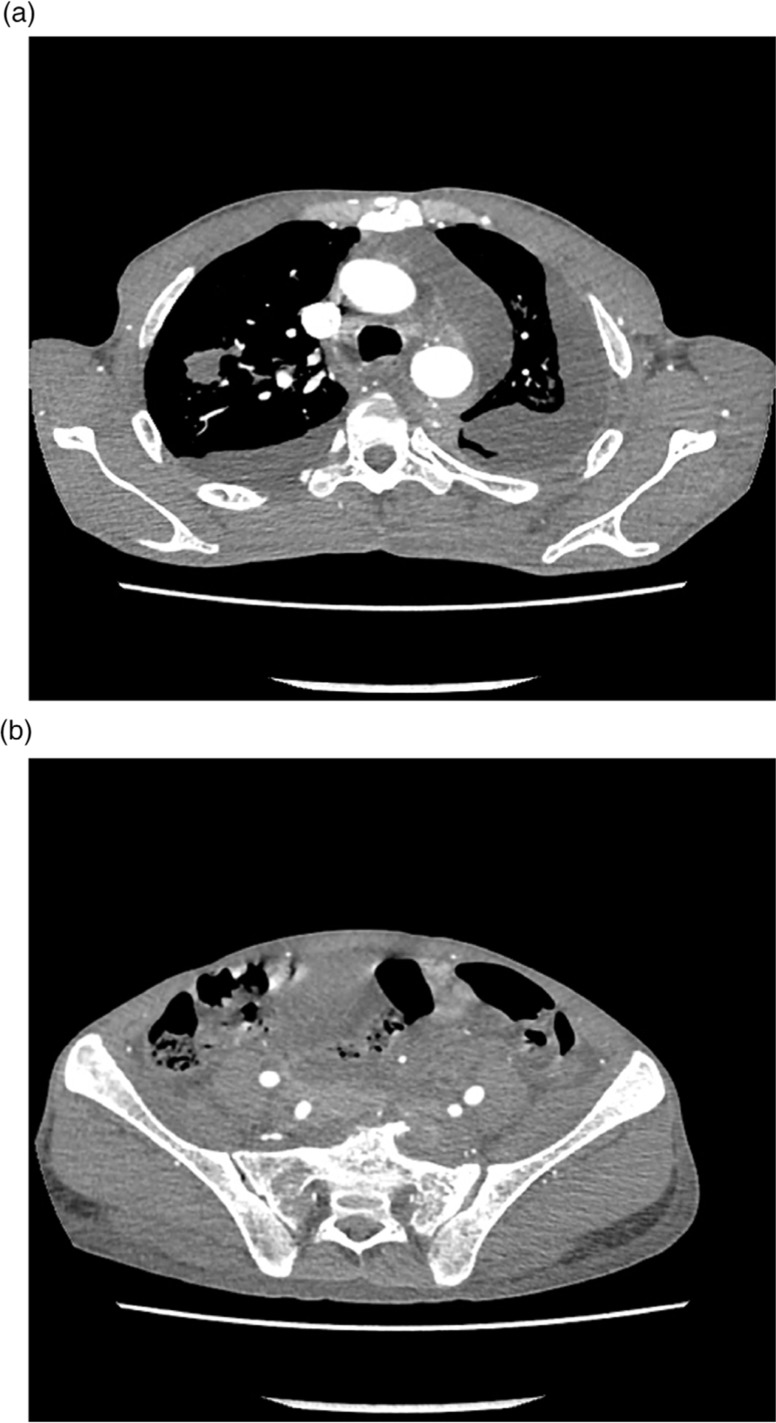
(a) Computed tomography scan of the chest with contrast showing bilateral effusion with pleural infiltration, right upper lobe involvement, soft tissue lesion in the posterior mediastinum. (b) Computed tomography scan of the abdomen showing lytic lesions in pelvis and sacrum.

**Fig. 4:**
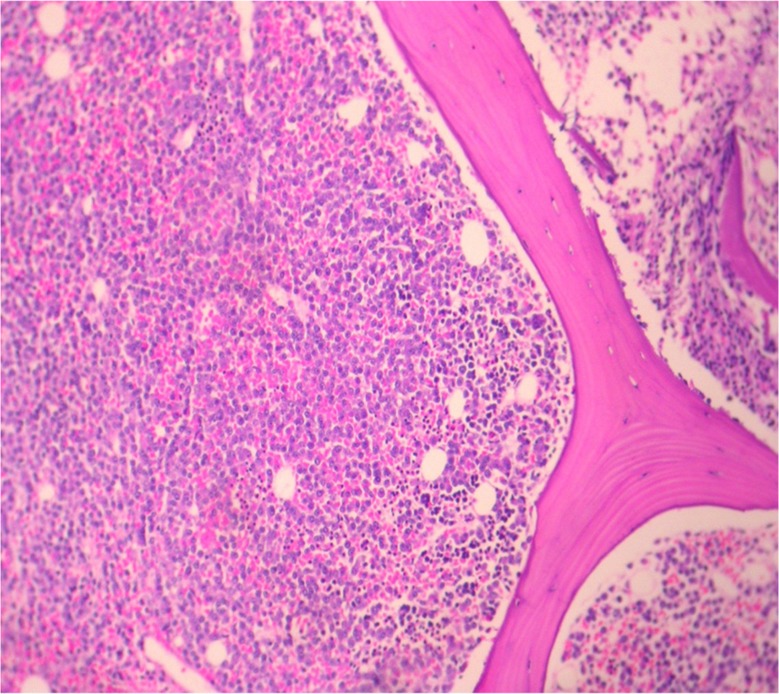
Bone marrow biopsy showing extensive replacement of marrow by sheets of atypical plasma cells, with binucleate and plasmablasts.

Laboratory analysis revealed the following results: white blood cell count: 4.9×10^3^/mm^3^ (84% neutrophils, 14% lymphocytes, 2% monocyte); hemoglobin: 8.8 g%; platelet count: 120×10^3^/mm^3^; erythrocyte sedimentation rate (ESR): 120 mm/h; total protein: 6.2 g/dl with albumin of 4.3 g/dl. Serum urea, creatinine, electrolytes, uric acid, calcium, lactate dehydrogenase (LDH), and liver enzymes were within normal limits. HIV serology was negative. A chest X-ray showed unilateral massive left side pleural effusion with mediastinal shift to right and right upper zone nodular opacity. It also revealed a small air pocket in the left apex, likely due to previous aspiration (Fig. 1). An intercostal chest drain was inserted at the emergency department due to severe respiratory distress and for both diagnostic and therapeutic reasons. Almost 3 L of hemorrhagic fluid was drained. Pleural fluid analysis revealed an exudative hemorrhagic effusion. The pleural fluid contained WBC 14,250 (35% neutrophils, 65% lymphocytes); RBC 0.10 ×10^6^/mm^3^; total protein 4.4 g/dl; LDH 663 IU/L. The results of bacterial and mycobacterial culture were negative. Cytological analysis of the fluid revealed numerous plasma cells including some binucleate forms and plasmablasts (Fig. 2). On echocardiographic examination, left ventricular ejection fraction was 60% with no pericardial effusion. Bence Jones protein was found to be positive in the urine. Serum protein electrophoresis did not demonstrate an M spike and immunoelectrophoresis showed polyclonal gammopathy. After 3 days of chest drainage a CT scan of the chest and abdomen was done, which showed bilateral pleural effusion more on the left side. There was patchy infiltration in the entire left lung and right upper lobe (Fig. 3a and b). There were also moderately enhancing low-density soft tissues lesions, seen extending from posterior mediastinum along the aorta, behind the diaphragm into the retro peritoneum. The left ureter was encased by the lesion causing mild hydronephrosis. There was also extension into the pelvis, producing significant presacral widening and encircling the recto sigmoid, prostate, and urinary bladder. Multiple lytic lesions were seen in pelvis and sacrum (Fig. 3b). These findings prompted a bone marrow biopsy that demonstrated extensive replacement of marrow by sheets of atypical plasma cells, many of which were immature, binucleate, and plasmablasts (Fig. 4). Immunohistochemically, the plasma cells were positive for CD 138 and Lambda light chain restricted suggesting a monoclonal malignant etiology. Based on the above features, a final diagnosis of PCM was made. The soft tissue lesion seen in the CT scan of the chest and abdomen was also concluded to be part of the same process. However, the patient did not undergo specific treatment for myeloma, and he left the hospital against medical advice.

## Discussion

Plasma cell myeloma is a plasma cell malignancy comprising about 1% of all malignancies as well as 10% of hematologic malignancies. PCM is a malignant proliferation of plasma cells which results in the production of monoclonal immunoglobulins (1, 3). This disease causes clinical signs and symptoms directly as a result of plasma cell infiltration and indirectly due to the effect of the abnormal paraprotein secreted. The most common presenting manifestations are fatigue, bone pains, and recurrent infections. Other less common, presenting symptoms, and signs involve renal, cardiac, musculoskeletal, gastroenterological, neurological, and respiratory systems. Thoracic manifestations commonly include osseous lesions, plasmacytoma, and rarely pulmonary infiltrates, mediastinal lymphadenopathy, pleural effusion (2). Rodriguez et al. reported the first case of MPE in 1994 (4).

Pleural myelomatous effusion is rare, occurring in less than 1% of patients during the course of the disease (1). The pathogenesis of myelomatous effusion is still unknown. Several possible mechanisms are postulated for myelomatous pleural effusion. The following mechanisms are possible: infiltration of the pleural fluid by malignant plasma cells, direct infiltration of the pleural fluid from adjacent tissues, nephrotic syndrome (secondary to renal tubular infiltration with paraprotein and the development of glomerular damage), pulmonary embolism, congestive heart failure secondary to amyloidosis, chronic renal failure, and mediastinal lymph node infiltration with lymphatic obstruction (2, 4, 5, 6). Of these several etiological factors, cardiac failure has been the most common cause for effusion in PCM. In our patient, the etiology may have been the direct infiltration of the malignant plasma cells to the pleura, as confirmed by the pleural fluid cytology and CT scan also showed infiltration of the pleura.

In the literature review, IgA myeloma was the most common isotope associated with malignant pleural effusion followed by IgG type, perhaps as a result of a major tendency to invade extra-osseous structures (1, 2). In a literature review of 958 cases of PCM in Mayo clinic, 58 cases had pleural effusion. However, pleural involvement secondary to myeloma was documented in only 0.8%. In this, the most common cause of MPE was congestive cardiac failure due to amyloidosis (6). Light chain myeloma can be missed on serum electrophoresis, and hence urine electrophoresis should always be done in the work up of PCM. Serum-free light chains should also be done in the initial evaluation of patients with suspected myeloma. As far as the diagnosis of myelomatous pleural effusion is concerned, several methods have been described. Pleural involvement can be diagnosed by the presence of plasma cells in pleural fluid or a pleural biopsy (3). Diagnostic criteria to confirm the myelomatous etiology are the detection of atypical plasma cells in pleural fluid cytology, demonstration of the monoclonal protein in pleural fluid electrophoresis (identical to serum protein electrophoresis), and histologic confirmation using a pleural biopsy specimen (4). Our patient met the first criteria, and medical thoracoscopy for histological confirmation was not done because of the patient's morbid condition. Pleural biopsy, urine electrophoresis, and light chain assay could not be done, as the patient left the hospital against medical advice.

The reported median survival time after the development of MPE is less than 4 months (2). The reason for shortened survival times in patients with MPE is unclear. At diagnosis the majority of patients were classified into advanced clinical stages and the laboratory characteristics of many patients indicated an aggressive clinical course. In addition, MPE represents clinical progression of the disease in itself. The mainstay of treatment is systemic chemotherapy. The MPE patients are usually resistant to treatment and often relapse in spite of aggressive systemic chemotherapy and further necessitating pleurodesis with chemotherapeutic agents (7).

The present case is unique and rare because the diagnosis was unsuspected in a young patient presenting with unilateral massive pleural effusion with pleural thickening, parenchymal lesions, osseous lesions, and extensive soft tissue involvement on radiological assessment.

In conclusion, the presence of atypical plasma cells in the pleura may provide useful evidence for the diagnosis of PCM. Myelomatous pleural effusion is a rare manifestation of the disease, as by itself it indicates an advanced stage of the disease and carries a poor prognosis. It should be considered when atypical plasma cells are seen in pleural fluid irrespective of age. Pleural fluid cytological examination continues to be an effective method to diagnose these cases and protein electrophoresis with immunofixation should be performed whenever possible.
